# Rewarding behavior change in rural communities: Pathways for a sustained inclusive change for child health

**DOI:** 10.1371/journal.pgph.0005273

**Published:** 2025-12-01

**Authors:** Shazwin Syed Ali, Farhana Tabassum, Zayaan Delawalla, Muhammad Asim, Mehwish Asad Memon, Mushtaq Mirani, Sana Khatoon, Zahra Ali Padhani, Imtiaz Sheikh, Muhammad Khan Jamali, Khan Muhammad Mangi, Zulfiqar A. Bhutta, Jai K. Das

**Affiliations:** 1 Rollins School of Public Health, Emory University, Atlanta, Georgia; 2 Institute for Global Health and Development, The Aga Khan University, Karachi, Pakistan; 3 Community Health Sciences, The Aga Khan University, Karachi, Pakistan; 4 School of Public Health, Faculty of Health and Medical Sciences, University of Adelaide, Adelaide, South Australia; 5 Robinson Research Institute, Faculty of Health and Medical Sciences, University of Adelaide, Adelaide, South Australia; 6 Centre for Global Child Health, The Hospital for Sick Children, Toronto, Canada; 7 Department of Paediatrics and Child Health, The Aga Khan University, Karachi, Pakistan; University of Washington Seattle Campus: University of Washington, UNITED STATES OF AMERICA

## Abstract

Worldwide, pneumonia, diarrhea, and malaria remain leading causes of death for children under the age of five, even though these diseases are preventable and can be treated. In public health, complex behavioral change interventions are often used. These interventions employ multi-component strategies and work on domains, such as education, policy, and environmental change, to promote prevention, control, and management of childhood diseases. In Tando Muhammad Khan (TMK), the Community Mobilization and Community Incentivization (CoMIC) trial employed a complex, participatory community engagement strategy and included conditional community-based incentives and showed promising results by improving child health related behaviors. A study was conducted to explore the experiences and perceptions of community members regarding the implementation and engagement processes of the CoMIC trial. A total of 13 IDIs and 16 FGDs were conducted to understand the factors behind the community engagement and behavior change that led to the success of the CoMIC trial and its adaptability for wider scale-up. The study identified four key motives for driving community engagement: egoism, altruism, collectivism, and principlism. The community’s close-knit social structure and shared sense of collective growth played a crucial role in encouraging participation and adapting to required behaviors. The trial focused on empowering the community by reinforcing health-seeking behaviors as a community responsibility, promoting cost-sharing to ensure long-term sustainability, and creating collective ownership through active community engagement and Conditional, Collective, Community-based Incentives (C3Is). By strengthening WASH and IYCF practices, increasing immunization uptake, promoting care-seeking behaviors, the intervention aimed to reduce the burden of childhood illnesses such as diarrhea, pneumonia, and malaria, ultimately improving child health outcomes.

## Introduction

Globally, pneumonia, diarrhea, and malaria are major causes of death among children under five years of age despite these being preventable and treatable [1]. Pakistan is one of the countries with the highest under five mortality rate [2]. Undernutrition, poor physical environment, and limited access to healthcare services contribute to high prevalence of diarrhea and pneumonia which are often referred to as diseases of poverty [3]. In 2013, the Integrated Global Action Plan for Pneumonia and Diarrhea (GAPPD) called for an integrated approach to address the challenges of pneumonia and diarrhea through interventions such as exclusive breastfeeding, appropriate complementary feeding, micronutrient supplementation, immunization, and improvement in water, hygiene, and sanitation (WASH) [4]. Despite the efforts of governmental and non-governmental programs, the uptake of these interventions remains low. This is largely due to health-related behaviors that are often resistant to change and influenced by various factors at the personal, household, and community levels [5].

Compliance with or adherence to proposed health practices is challenging, as it requires changing lifestyle behaviors that are deeply rooted in environmental, demographic, and personal factors [6]. Behavioral sciences focus on building resilience among individuals and the community by using capabilities and opportunities, such as community resources, social capital, and leadership, to integrate desired health practices into their lives [7]. The intention to act is an immediate precursor to behavior and requires strong behavioral control, which arises from the right opportunities [8]. However, these opportunities are also shaped by broader structural factors, such as socioeconomic inequalities, healthcare accessibility, and policies [9]. Therefore, a well-planned behavioral change intervention has two key components: the desired behavior outcomes and the methods used to deliver behavioral change techniques (BCTs) [10].

Complex behavioral change interventions have been widely used to address public health issues including prevention, control, and management of childhood illnesses. A cluster randomized trial was conducted in Lusaka, Zambia, employing multiple behavioral change interventions for diarrhea control among the caregivers of children under the age of five [11]. This was called the Komboni Housewives intervention, which ran in 16 health centers for six months. The intervention leveraged emotional drivers, such as social acceptance, inclusion, validation, agency, and empowerment. Program activities included clinic sessions with monthly prize draws, community-based women’s forums, roadshows, and radio messaging. The primary outcomes of this intervention included improved behaviors around exclusive breastfeeding, personal hygiene, and oral rehydration in diarrhea treatment, measured using self-reports, observations, and demonstrated practices compared against standard care in the control group [11]. A 35-month radio campaign intervention in rural Burkina Faso targeted towards women and caregivers of children under the age of five reported improvements in child survival-related behaviors, including increased care-seeking and timely treatment for different illnesses. 14 geographic areas were chosen based on their high listenership, seven areas were randomly assigned to the intervention arm, and the other seven to the control arm. The radio messages of 1-minute duration and long-format programs of 2 hours are broadcast five days a week. The content covered two targeted behaviors per day. Self-reported care seeking for different childhood diseases, especially diarrhea, increased between baseline and midline [12].

Similarly, the Community Mobilization and Community Incentivization (CoMIC) trial aimed to improve the uptake and adherence to recommended preventive and curative practices for childhood diarrhea and pneumonia in rural Pakistan. This study aims to explore the experiences and perceptions of community members regarding the implementation and engagement processes of the CoMIC trial. Findings of the intervention carry important implications for policy makers to prioritize community-led incentive models, for program implementers to integrate cost-sharing and collective ownership mechanisms, and for broader scalability by adapting this approach in other rural, resource-constrained settings.

## Methodology

This qualitative study was conducted to evaluate and understand the experiences of the community members involved with the CoMIC cluster randomized control trial (NCT03594279) [13].

The study brings forward in-depth insights from the community on the intervention outcomes, processes, and potential areas of improvement, such as the positive community response to incentivization, increased participation in health interventions, willingness to change practices around WASH, childhood nutrition, vaccines, management of diarrhea, and pneumonia in children, and addressing infrastructural limitations for promoting healthy behavior changes. Additionally, the study highlights challenges related to healthcare accessibility, its gaps, and barriers to vaccine coverage and its solutions.

The summative study took place from 01/12/2020–31/03/2021 in the district of TMK, situated in the province of Sindh, Pakistan. The administrative district has an area of 1,423km^2^ and a population of 726,119 [14]. This district has the highest poverty level (89.3%) across the 29 districts of Sindh [14]. The district comprises three administrative talukas and 17 union councils (UCs) with a population density of about 475/ people per km^2^ and an annual population growth rate of 2.3% [14].

### Primary study

The CoMIC trial was a three-arm cluster randomized trial with two intervention groups and a control group conducted between 01/10/2018–31/10/2020 [13]. The trial evaluated the effectiveness of a unique approach to community engagement and demand creation through mobilization and incentivization aimed at improving the uptake of essential interventions for the prevention and management of diarrhea and pneumonia, the two major causes of morbidity and mortality among children under the age of five years. A formative study conducted before the implementation of the trial showed overwhelming support for community groups and C3Is. However, it also identified a significant lack of information regarding childhood diseases and poor immunization rates, attributed to limited coverage by health workers and community’s inability to travel outside the village for healthcare [15]. The two intervention arms, namely the Community Mobilization (CM) and Community Mobilization and Incentive (CMI) contained the community mobilization component of the two intervention arms focused on forming of Village Committees (VCs) comprised of Union Council members, local leaders, religious leaders, and other prominent male and female members of the community [16]. These individuals first participated in training sessions that equipped them to lead activities. Activities included from door-to-door visits and group meetings aimed at raising awareness and motivating the community on issues related to childhood nutrition, WASH, vaccines, management of diarrhea, and pneumonia in children. Additionally, these group sessions also involved the identification of community level health, nutrition, and sanitation problems, as well as developing locally feasible strategies to address them. For the CMI arm, incentives were provided if the clusters (expanded below) met their required targets.

The intervention included community-based incentives (C3Is), which were collective conditional non-cash rewards for clusters that met targets to improve composite coverage of child health interventions—immunizations, ORS use, and sanitation. Targets were 10% improvement at 6 months, 25% at 15 months, and 50% at 24 months. Incentives, mainly water and sanitation facilities, were decided by village councils with community input to ensure fairness and shared benefits [17].

The thresholds were set to promote feasible, gradual behavior change, starting with easier practices and moving toward sustained habits. Composite coverage was the average of immunization rates, ORS use, and sanitation scores, linking incentives to overall community health improvements [17].

### Participants and recruitment

The CoMIC research team identified potential stakeholders for the Focus Group Discussions (FGDs) and In-depth detailed interviews (IDIs) through a purposive sampling technique. This sampling technique facilitated the recruitment of a mixed set of participants from our intervention clusters based on gender and their key roles within the community outlined in the participants categories (Table 1). These participant categories included parents, community elders, healthcare providers, Union Council representatives, teachers, doctors, and vaccinators from intervention clusters. Furthermore, interviews were also conducted with the District Health Officer, focal persons for vaccinators and Lady Health Workers (LHWs), and the Assistant District Commissioner (Table 1).

**Table 1:** Description of study participants in both incentive and mobilization clusters.

FGDs	CM	CMI	Total FGDs conducted
Mothers	2	2	4
Fathers	2	2	4
Community Elders	2	2	4
Healthcare Providers (HCPs)	2	2	4
	**Total FGDs (n = total number of participants)**		**16 (n = 160)**
**IDIs**	**CM**	**CMI**	**Total IDIs conducted**
UC Councilor/ Chairman	1	2	3
School Teacher	1	1	2
Doctor	1	1	2
Vaccinator	1	1	2
Focal Person Vaccinator	–	–	1
Focal Person LHW	–	–	1
Assistant District Commissioner	–	–	1
District Health Officer	–	–	1
	**Total IDIs**		13
	**Grand Total**		**29**

### Study guide

The research team used evidence from existing literature on community engagement and incentivization and assistance from our prior qualitative interview guide for the CoMIC formative study. Semi-structured interview guides were developed for each stakeholder group in the English language. The interview guides were reviewed by senior researchers in public health and social sciences. After incorporating the feedback, the guides were translated into the local Sindhi language. The guides included questions on program evaluation in the areas of community mobilization, incentives, the status of healthcare, health practices related to immunization, Infant and Young Child Feeding Practices (IYCF), WASH, and childhood diseases, and program sustainability.

### Data collection

The interviews were conducted by trained qualitative researchers familiar with the context and language spoken by the community. Each IDI and FGD was approximately between 40–60 minutes and 50–90 minutes, respectively. The interviews were conducted in the local Sindhi language and were recorded after taking the written consent using a digital voice recorder. Detailed field notes were also taken during each interview which then were compiled together for a rapid analysis. Field notes and audio recordings were transcribed into Sindhi and then translated into English by the bilingual translators. All transcripts were checked for quality and stored under pseudonyms.

### Data analysis

Thematic analysis was conducted using NVivo software (version 10) [18] to arrange and code the transcribed interviews. The codebook was developed iteratively by SA and FT using a combination of inductive and deductive approaches. The inductive approach allowed themes to emerge directly from the data, while the deductive approach was informed by the themes and theoretical constructs that emerged during formative qualitative research. Braun and Clarke’s thematic approach has been used [19]. The research team read the transcripts multiple times to familiarize themselves with the content and context. Primary codes were generated by SA and reviewed by FT which then were rearranged into thematic areas. Sub-themes were then categorized into grand themes and renamed to make them more specific and relevant. In the end, three major themes; 1) motives behind community mobilization, 2) program impact on health practices, and 3) multifold benefits of community-based incentivization, were finalized with input from MA and JD. Using an inductive approach, transcripts were coded and recurring themes, such as selfless community services and ethical obligations surfaced directly from the data when describing the qualities of VCs and their role in community engagement. These qualities were then categorized under altruism and principlism, respectively. Likewise, deductive lens was applied using insights from summative qualitative research which highlighted egoism and collectivism as relevant thematical constructs. These four elements; Altruism, Principlism, Egoism, and Collectivism, were recognized as drivers of community mobilization [20].

We ensured the rigor of analysis through multiple forms of triangulation. The source triangulation was achieved through interviews with diverse stakeholder groups, including local community members, government officials, and healthcare professionals. Methodological triangulation was achieved by combining multiple qualitative data collection methods, such as FGDs and IDs. These triangulation measures verify consistency and strengthen the credibility of our findings.

### Ethical consideration

Ethical approval was obtained from Aga Khan University’s Ethical Review Committee (ERC number: 4676-WCH-ERC-17). The research participants from the community were informed about the study objectives and their participation was consented to and voluntary. Through the written informed consent form, the participants were explained about their rights to refuse questions or leave the interview at any point as well as having the right to ask questions before, during, and after the interview. All interviews were conducted in spaces recommended by the participants themselves. The confidentiality of participants’ identities was ensured by employing auto-generated participant codes in the place of their names.

**Table 2:** Characteristics of the study participants.

Focus group discussions (N = 16)	
Variable	N%
Parents	
Mothers	30(50.84)
Fathers	29(49.16)
Community Elders	
Male	14(50.00)
Female	14(50.00)
Age	37.35 ± 8.00*
Education	
Illiterate	27(21.43)
Primary education	23(18.25)
Middle education	16(12.70)
Secondary education	28(22.22)
Higher secondary education	20(15.87)
Higher education	12(9.52)
Occupation	
Skilled	65(52.00)
Unskilled	17(13.60)
Housewife	43(34.40)
**In-Depth Interviews (N = 13)**	
Year of experience	14.83 ± 11.48*
Age	46.69 ± 10.61*
Qualification	
Basic Education	4(30.77)
Higher Education	9(69.23)

*mean ± standard deviation.

## Results

**Fig 1:**
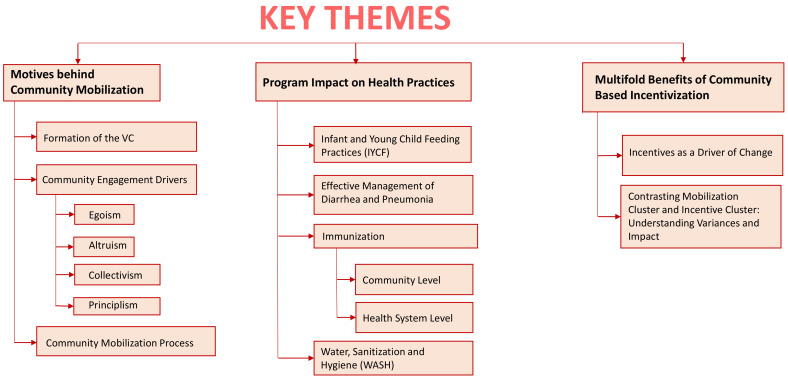
Key themes presented in our findings.

The result section presents key themes (Fig 1) that emerged from the analysis of 13 IDIs and 16 FGDs (Table 2). The following themes were identified: motives behind community mobilization, program impact on health practices, and multifold benefits of community-based incentivization.

### Motives behind community mobilization

This theme provides insights into the driving forces that prompted community members to engage in community mobilization efforts. This section delves into the details of community mobilization activities, beginning with how the VC was formed, the four community engagement drivers, and the process of mobilizing the community.

#### Formation of the VC.

Community mobilization and participatory decision-making proved to be pragmatic choices for rural communities because of the trust, social consciousness, and strong social network among the members. The formation of VCs exhibited great satisfaction and acceptance from the community with an understanding of why some people deserved to be in the VC more than others. Intelligence, proactive attitude, confidence, good articulation, influence, responsibility, awareness and familiarity with the community’s problems, commitment, and willingness were the qualities that were assumed to be held by a VC member.

However, initial formation of the VC was met with hesitation, as some community members feared that promised incentives might be delayed or not delivered. This created uncertainty around whether joining the VC would truly benefit the community. Over time, through a series of meetings and visible progress, trust was gradually built and members expressed satisfaction with their involvement in the decision-making process.

*“A less aware person has no idea, but an astute member will take charge, and other people can also benefit from his guidance.” (FGD, Fathers, Mobilization Cluster)*

*“We are fortunate that they are skilled at speaking. They are dedicated to the community and can talk to people nicely. When a senior officer visits, they are able to communicate well with him.” (FGD, Fathers, Mobilization Cluster)*

*“They must know what to do and how to handle everything. It doesn’t matter if they are not literate, they must be active.” (FGD, Female, Community Elder, Incentive Cluster)*

### Community engagement drivers

While the above-mentioned preference or criteria existed for a VC member, the selection was also open to anyone willing to be part of the VC. We found that people joining the VCs and the community participating in the CoMIC activities revolved around one of four motives: egoism, altruism, collectivism, and principlism. These four motives that drove community mobilizers to rally behind shared goal of improving the quality of life of the community. When individuals find meaning and value in their actions, they are more likely to continue these behaviors, creating a foundation for long-term commitment to community-driven change.

### Egoism

Those individuals wanting to enhance social standing in their community joined the VCs because of egoism. These individuals were focused on achieving recognition and building a positive reputation within the community. The quote from a VC member mentioned below shows that engaging with others sometimes means advancing one’s own aspirations. While there is collective participation, there is emphasis on a personal sense of pride and recognition derived from that involvement.

*“Right now, there are 8 or 9 women working together, and it satisfies me. It motivates me to move forward. Now, I feel honored. I thought well of all of them. I stand in front of them, and I will stand with them in the future. Together, we will be able to achieve something meaningful.” (FGD, Mothers, Incentive Cluster)*

### Altruism

Many participants expressed altruism in their reasons for participating in the trial activities. Demonstrating a strong desire for the welfare of others, they selflessly contributed their time and efforts to support the community’s progress. The VC members became the face of the CoMIC trial for the community. They were often friends, neighbors, relatives, or family members of the people in the community which encouraged trust and credibility towards the VC, and the community members became readily receptive and naturally approved of the series of behavioral change activities. Shared realities, values, and a common understanding of the local context motivated the VC members to selflessly dedicate their time and energy to improving the social conditions of their people.

*“I get up early in the morning, pray Fajr, and go to the village. I ask my villagers to get up, clean, sweep, and wash their utensils. I’m just a poor farmer. I don’t have a salary, but I have an interest in this social work. It is okay if I do not get anything for it, but from my actions, if someone gets any benefit, then this would please me.” (IDI, UC Councilor, Incentive Cluster)*

### Collectivism

The spirit of collectivism was prominent among the community. People were united to work toward shared goals and benefits. The collaborative approach allowed participants to pool their resources and efforts to improve the overall conditions in their community.

*“If anyone leaves or is unable to participate due to household chores, the group visits them again. Sometimes, they [women in community] would set aside their own work to attend a meeting.” ((FGD, Mothers, Incentive Cluster)*

*“We had no prior knowledge or information, not even the concept of vaccination. But when the CoMIC team came to us, we started having meetings and forming committees. Together, we put in a lot of effort, and now, Alhamdulillah, we have seen some improvement.” (IDI, UC Councilor, Incentive Cluster)*

### Principlism

Last, many individuals were driven by a sense of moral responsibility. Principlism guided their actions, as they adhered to ethical values and sought to make decisions that aligned with these principles.

*“We have a responsibility to make sure that what we received from this program, we give it forward to others. The benefit that we have gotten from the CoMIC team, now let’s give this benefit to people in those villages where there is no awareness. We can tell them how these things are facilities for you to keep clean. Keep ORS at home, it prevents your children from diarrhea and get vaccinated. It’s all for our benefit.” (FGD, Fathers, Incentive Cluster)*

### Community mobilization process

The community members exhibited social cohesiveness through their words and actions. Even with limited access to communication technology, the community in TMK had a powerful tool for spreading information, that is, word of mouth. The health information delivered to the VCs was, with much responsibility, spread across other people because the community flourishes on the idea of collective growth.

*“I sat with [name of LHW] then we gathered all women together and began the mobilization. For example, eight to nine women are sitting in the session and if the information is understood by them and they are satisfied then this chain will move forward.” (FGD, Female Community Elder, Incentive Cluster)*

Besides information sessions led by the VC members in the villages, the VC members also actively ensured compliance with the delivered health messages. The efforts put in by the VC members to visit households to observe and guide them on healthy lifestyle choices and practices enhance behavioral change.

*“We visited the houses in villages to see if there was animal dung in their living area or if the kitchen was not properly cleaned. From time to time, we visited the houses, those who were young or less aware, we taught them that if you keep clean you will get considerable benefits.” (IDI, Teacher, Incentive Cluster)*

### Program impact on health practices

The key focused areas – Infant and Young Child Feeding Practices (IYCF), childhood diseases, immunization, and Water, Sanitation, and Hygiene (WASH) – are informed by the formative research. The formative phase provided detailed insights into the community’s social structure, norms, and needs. Gaps in health literacy, access to healthcare services, childhood disease management, and adequate WASH facilities significantly dominated the community characteristics. These characteristics were not only pivotal in informing the incentivization and mobilization efforts but also helped identify the priority areas central to evaluating the program’s impact on health outcomes.

Stakeholder groups brought different perspectives that enriched the understanding of changing health practices. Community members, particularly women, more often described the observable practices at the household level, such as changes in hygiene routine and child feeding practices. In contrast, men and health staff brought insights about broader system-level and community-level dynamics, offering reasons why certain barriers persisted and what structural issues inhibit the uptake of practices.

### Infant and young child feeding practices (IYCF)

A significant improvement was observed among the community, especially women, regarding breastfeeding practices. The female participants could reflect on the misinformed practices and were able to distinguish between their previous knowledge and practices and what had been learned through the CoMIC intervention. Findings revealed early initiation of complementary feeding. The community members, including mothers, believed that they were not producing enough milk to satisfy the needs of the newborn, because of which, top feeding was widely practiced. These misconceptions and practices were addressed through raising awareness among mothers about the benefits of exclusive breastfeeding and optimal infant feeding practices to improve child health.

*“It was said that mothers cannot provide sufficient milk as per the child’s need, that’s why complementary feeding should be done. We now know that the mother should eat well and be nourished so that adequate milk can be produced.” (FGD, Community Elder, Incentive Cluster)*

Some commonly given substitutes for breast milk include flour mixed with water, grape water, honey, butter, water, goat milk, and buffalo milk. These misconceptions were addressed by emphasizing the importance of exclusive breastfeeding for a baby’s first six months.

*“Even a child as old as 8 days was given water mixed with wheat flour. I used this practice for my elder son and daughter. We did not know back then but now we know that only mother’s milk must be given to the babies.” (IDI, UC Counsellor, Incentive Cluster)*

*“Three to four years ago, our old ladies used to give grape water and other traditional drinks to babies. People from the CoMIC team told us to give mother’s milk immediately to the baby after birth and it should be the sole source of food for the infant.” (FGD, Community Elder, Mobilization Cluster)*

Colostrum has been surrounded by a lot of misconceptions in the community. It was viewed as harmful or impure, leading to the practice of discarding it after childbirth. However, the awareness efforts as part of the intervention activities were able to change these views about colostrum. The community was informed about the benefits of colostrum for newborn’s immunity and overall health.

*“The first milk of a mother which is yellow in color (colostrum) must be given to the child. It was previously discarded and not given to the child.” (IDI, Teacher, Mobilization Cluster)*

*“Previously, it was believed that this milk (colostrum) since it is yellow, is bad and should not be given to the baby. The CoMIC team came to us and told us that this should not be discarded as it strengthens the baby’s bones.” (FGD, Mother, Mobilization Cluster)*

For several years, doctors and LHWs have made efforts to address the misguided practices related to IYCF through health education and promotion activities among the communities, however, a need for further improvement in IYCF practices was required. Community mobilization activities as a part of the CoMIC trial involved elders and community influencers and played a far-reaching effect on the community in terms of acceptability and improved health behavior. As a result of health information dissemination in the CoMIC trial, the participants exhibited consistent information on the IYCF practices. Additionally, previous misconceptions were also clarified which allowed the participants to logically reason the claims.

### Effective management of diarrhea and pneumonia

The lived experience of common childhood diseases, such as diarrhea and pneumonia, has already familiarized the community with its symptoms, causes, and preventive measures. In addition, the primary healthcare providers, including the LHWs and other local NGOs, have attempted to improve people’s knowledge and health behaviors. However, false perceptions and poor health practices regarding various childhood diseases persist, which calls for further health counseling. As part of the CoMIC trial, trained village committee members were involved in information dissemination. The qualitative findings indicate improvement in community knowledge with similar understanding levels noticeable among all participants. The community identified poor sanitation and hygiene practices and lack of vaccination as the main cause of diseases among children.

*“Where there is a baby playing, there’s a washroom, at the same place the baby is eating, at the same place he is defecating…kitchen, washroom everything is in one place. Unhygienic conditions give birth to diseases.” (FGD, HCP, Mobilization Cluster)*

ORS is a crucial component in the management of diarrhea. Awareness of and acceptance of the use of ORS has been improved. Since the trial, the availability of ORS in the stores has also improved. Simultaneously, the people are taught a home-made drink using common ingredients that can act as a substitute for ORS, in case of its unavailability.

*“Previously, ORS was not available at shops. But now it is available as people now have a demand for it.” (FGD, Community Elder, Incentive Cluster)*

The HCPs shared about treating pneumonia with antibiotics and nebulization. At the household level, applying balm or betel (paan) leaves on the child and wrapping the baby in a warm cloth, taking hot drinks, and taking steam were the immediate initiatives taken for pneumonia management.

### Immunization

The CoMIC trial has vastly impacted immunization trends and practices in TMK. Before the trial, many people already recognized the benefits of vaccination and showcased positive health behavior, in terms of, willingness and adherence to vaccination. Whereas, a few people, especially the poor, did not know about the role of vaccines. Nevertheless, the effectiveness of immunization remained questionable in TMK due to partial or delayed vaccination because of a lack of awareness about the vaccine schedule and the importance of persistence in immunization. Poor or incomplete knowledge in combination with other structural challenges, such as distance from health facilities, and vaccine hesitancy, continued to contribute to poor immunization outcomes. The CoMIC trial recognized the need to improve knowledge and address community concerns and fears regarding vaccination.

### Community level

The participants were surprised to learn about the many benefits of vaccination and how persistence or compliance with vaccine schedules and intervals is crucial for vaccine effectiveness.

*“As a river bridge protects us from sinking in the water. Similarly, the vaccine protects our children from many diseases.” (FGD, Community Elder, Mobilization Cluster)*

Moreover, the participants were able to recall quite a few vaccine-preventable diseases.

*Vaccines do not just give immediate health benefits; their benefits prevail for a lifetime. They protect children against major diseases even when they grow up, such as Hepatitis C, pneumonia, or diarrhea. We now know the names of the vaccines, so we inquire about the availability of an appropriate time to vaccinate.” (FGD, Community Elder, Mobilization Cluster)*

Earlier, people did not know the purpose of the immunization cards that are provided free of cost from the health facility at the time of birth or first vaccine. Our intervention ensured that the community could comprehend the details on the card. Participants expressed their satisfaction in being able to keep a record of the next vaccination time for their children through the card.

*“When we vaccinated our children, we did not keep their cards, but your program brought our attention to keeping a record of vaccination through these cards.” (FGD, Community Elder, Incentive Cluster)*

### Health system level

Health infrastructural limitations also play an important role in vaccine coverage. The CoMIC trial mediated communication gaps between the community and HCPs.

*“No one came to us before your program. Only a few percent of people used to take their children for vaccination…your program called vaccinators to the village and now they continue to come here.” (FGD, Community Elder, Incentive Cluster)*

*“Your program started a series of health sessions in which doctors or other health staff visit the villages and meet people and notable persons of the community. A good relationship and liaison developed between health care providers and the community. For instance, there is a village where your program’s team member took me once to conduct sessions. Now if the people from that village face any problem, they call us directly that’s why this series should be continued.” (IDI, Doctor, Incentive Cluster)*

Despite the actions undertaken by the CoMIC trial to improve vaccine coverage and child health, some barriers at the structural level and resistance at the behavioral level persist (Table 3).

**Table 3:** Barriers to vaccine coverage and its solutions.

Barriers	Solutions
Low awareness	Continuation and expansion of community mobilization activities in liaison with the HCPs.
Proximity to health facilities, travel costs, and conveyance issues	Facilitate the distanced population from the health facilities through the LHW network and outreach vaccinators
Vaccine hesitancy – fear of infertility, fever, and other cultural beliefs	Health education and promotion activities must be conducted to dispel the misconceptions and fears held by the community related to vaccines. Provide or prescribe Panadol drops after vaccination to control fever and improve the community’s trust in vaccines. *“It would be better for the provision of vaccines to also be facilitated with Panadol drops. By this communities’ trust will remain intact.” (IDI, Vaccinator, Mobilization Cluster)*

### Water, sanitation, and hygiene (WASH)

TMK experiences several issues concerning WASH which include limited access to clean water sources, inadequate sanitation facilities, lack of infrastructure and resources to facilitate WASH, and poor personal hygiene (Table 4). In the initial stage of the trial, upon asking about their community-based needs, the people were imperative to have the water system fixed for their benefit. The CoMIC trial supported the communities in TMK by enhancing their knowledge of hygiene practices and methods to ensure the safety of drinking water. Moreover, the intervention shared the costs of installing drinking water and sanitation facilities provided to various communities in TMK. After three to four years, it was observed that although awareness and hygiene practices have improved, the infrastructural challenges persist. Structural barriers, such as lack of space for latrines and poor ventilation in household washrooms, further restricted consistent use of facilities. For instance, although open defecation has decreased due to the building of latrines and washrooms, the use of these services has only become more gendered. In some cases, women find it shameful to use washrooms in the presence of male members in the house, which restricts women’s use of washrooms. In addition, religious barriers were noted, as some Hindu households avoided constructing washrooms inside homes out of respect for their deities. It shows that despite the community’s willingness to change its practices around WASH, the infrastructural limitations do not allow for a healthy change (Table 5).

**Table 4:** WASH challenges.

Limited access to clean water sources
*“We travel 2-3 kilometers to fetch water.” (FGD, Community Elders, Female, Incentive Cluster)*
*“Water comes from inappropriate sources, impurities float in that water. There is no filtration, and this water is directly consumed by the people due to which abdominal diseases like diarrhea happen.” (IDI, Vaccinator, Mobilization Cluster)*
**Inadequate sanitation facilities**
*“You will find communities where washrooms are not available. They will defecate outside in agricultural fields which causes foul smell and serious diseases” (IDI, Vaccinator, Mobilization Cluster)*
*There are only bushes. Men get up early in the morning, take their water vessel (lota) and go in the open for defecation.“(FGD, Fathers, Mobilization Cluster)*
**Lack of infrastructure and resources**
*There is no system to dispose waste, but after being told by the program team we now dig pits in the ground or safely burn it in the bins. We nor have any drainage system.“(IDI, UC Councilor, Incentive Cluster)*
*Now the thing is that the program has given us washrooms, but we don’t have a drainage system anywhere.“(IDI, Teacher, Incentive Cluster)*

**Table 5:** Previous WASH practices and behavioral change at environmental, household, and individual levels.

Previous practices	Behavioral change	Quotes
**Environmental Level**		
**Open Defecation** Most people, especially the poorer communities practice open defecation	Construction of washrooms	*“The facilities of washrooms are now available. There are no more flies and smell.” (FGD, Community Elders, Female, Incentive Cluster)* *“We now have washrooms through the intervention incentives otherwise people used to defecate in the open fields.” (FGD, HCPs, Incentive Cluster)*
**Absence of drainage system** Some people who had washrooms installed in their homes faced the challenge of having no drainage system.	Considering the infrastructural limitations, people learned alternate human waste management practices, such as constructing pits, filling them with calcium carbonate, and covering them.	*“The CoMIC team gave us the material and we built the washrooms. We dig a pit and fill it up with calcium carbonate and cover it with a lid so that germs do not spread.” (FGD, Community Elders, Female, Incentive Cluster)*
**Poor waste management** Littering streets, foul smell, flies, and the spread of diseases	Putting garbage in pits and covering or burning it in bins	*“Previously, our ladies threw the garbage on the streets. Now when the CoMIC team came, they said to put in pits or burn it on the other side of the site.” (FGD, Fathers, Incentive Cluster)*
**Household Level**		
**Human exposure to unclean animals and their living areas** Keeping cattle indoors, not cleaning the cattle and neglecting the cleanliness of cattle and their living area, children playing with cattle and roaming barefoot in their living area, and sharing eating utensils between animals and people	Animals are now kept away from living areas, animals are washed, and utensils separated, limiting children’s contact with animals, and ensuring children’s hygiene after interacting with animals.	*The cattle used to be with us at home, but since your people told us about cleanliness, we keep the animals outside. Previously, people and animals lived together, but now we have separated our living areas.” (FGD, Community Elders, Female, Incentive Cluster)*
**Poor household hygiene**	Improved household hygiene as a result of awareness and incentivization	*“When we cook, we wash our hands before it. We keep our meal and water pots covered at safe places.” (FGD, Mothers, Incentive Cluster)*
**Individual Level**		
**Poor personal hygiene**	Use of soap for handwashing after using the toilet, before eating, and before cooking	*“We didn’t wash our hands with soap, but now we do.” (FGD, Mothers, Incentive Cluster)*

The WASH facilities provided by the intervention leave room for further expansion of consistent efforts. However, it is important to understand the factors that restrict the community from fully benefiting from the available WASH facilities (Table 6).

**Table 6:** Factors inhibiting the uptake of WASH facilities.

Social Barrier	*“Recently I visited a community where I asked about the new washrooms, they said that we don’t feel comfortable going to or using washroom when male family members are around…so the secret of not using washroom is that they feel shy in front of their male family members.” (FGD, HCPs, Incentive Cluster)*
Religious Barrier	*“Hindu community members usually say that our gods or temples are at our homes, they respect their gods that’s why they don’t build washrooms in their homes.” (FGD, HCPs, Incentive Cluster)*
Physical Barrier	*“People do not have a habit of using washrooms. They complain of having difficulty sitting there or feeling shortness of breath, and suffocation because of closed door.” (FGD, HCPs, Incentive Cluster)*

### Multifold benefits of community-based incentivization

This theme brings insights from the community members as part of the incentive cluster. The C3Is delivered through the CoMIC trial encourage ownership and empowerment by giving them a chance to become drivers of change as well as the ones benefiting from the change. The community members expressed interest and agreed to contribute towards labor and land provision.

*“The CoMIC team only provided the building material for the washroom; the wages and labor charges were borne by the people.” (IDI, Teacher, Incentive Cluster)*

Awareness coupled with C3Is enhanced people’s capability to be able to afford and pursue healthy lifestyle choices. The benefit of this incentivization strategy was not only limited to the beneficiaries, but it extended to inspire others to adopt healthier behaviors or pursue similar health-focused initiatives. Incentives acted as an external validation of the short-term behavioral changes. It means that participants engaged with the promoted behavioral changes and their motivations were not only by willingness but also by the incentive structure tied to achieving specific performance metrics. Furthermore, by having both a collaborative decision-making process and involving the community in the operationalization of the incentives, there was an innate sense of pride and ownership that emerged within those responsible for increasing the quality of life of their families and their community members.

*“They give us knowledge. We learned lessons from it. Some are cleaning their village while others are building bathrooms on their own.” (FGD, Male Community Elder, Incentive Cluster)*

### Incentives as driver of change

The section discusses the decision-making process regarding selecting an appropriate WASH facility based on community needs and fairness to those eligible for incentives. It also discusses the health impacts and perceived benefits of WASH-based incentives. Moreover, it is important to note that short-term tangible incentives served as drivers of change within our intervention communities.

This was achieved by recognizing the importance of the construction of toilets and latrines and the improvement in quality of life through the installation of hand pumps Not only did the community show eagerness to receive C3Is, but they were also familiar with the project objective and eligibility criteria for receiving incentives and made an active effort to change their hygiene and sanitation practices, vaccinate their children, and improve the uptake of essential interventions for diarrhea and pneumonia.

Despite their eagerness, some respondents initially expressed concern about delays in receiving incentives, which they feared might undermine the fairness of the process. These doubts were addressed through transparent communication and open community meetings, which helped reassure members that the distribution would follow agreed rules and timelines.

The decision-making process was observed at a smaller group level followed by a community-level discussion. Targeting preferences were mutually decided between the VCs and the community through discussion. The community members considered the distribution of incentives fair by virtue of having the community lead the conversation about the appropriate incentives – allowing them agency over their power to influence and take ownership of these incentives. Furthermore, the cost-sharing agreement furthered their sense of ownership and improved their role as active stakeholders in the project, leading to long-term sustainability of the incentives.

*“People from all the villages come together for the meeting. Hiding anything is not possible as the discussion is open to all. The distribution is based on merit. Many needy people have met the criteria, but it is on the meeting participants to decide to whom the washroom will be provided in this round and who may receive it in the next.” (FGD, Male Community Elder, Incentive Cluster)*

Based on the discussion and consultation with the VC members, the project team devised a composite indicator to determine eligibility for beneficiaries. Further prioritization, based on the sequence of getting incentives, was done by the community. Interestingly, the community exhibited a complex cognition behind determining the sequence of incentive distribution based on their unique understanding of the need and vulnerability. Due to the close-knit structure of our intervention clusters, respondents were cognizant of the needs of their neighbors, leading to an equitable distribution, with the impact of the incentives becoming greater than the sum of its parts.

Ultimately, the gradual resolution of these fears and delays strengthened trust in the process. Community members came to see their role in the VC not only as linked to incentives but also as central to achieving the broader objectives of improving child health and living conditions through collective action.

*“There are some who live in mud houses and others who do not even have a house – they live in the out in the open. Then some are orphans and there are those households that do not have a male member. Based on these factors we then decide in favor of these people.” (FGD, Female Community Elder, Incentive Cluster)*

*“We asked our neighbors for advice, then call them, and we gave incentives to those who are in need. We choose one of them and give it to them.” (FGD, Male Community Elder, Incentive Cluster)*

### Contrasting mobilization cluster and incentive cluster: understanding variances and impact

The qualitative insights gathered from the participants reveal that the effectiveness of community mobilization fades with the course of time for two main reasons: lack of motivation and insufficient resources to put the learned concepts into practice. It was observed that with the provision of C3Is, not only the community engagement and mobilization remain continuous, but the possible utility of the provided WASH facilities enhanced the community’s capability to put the acquired knowledge into action.

The participants from the mobilization clusters showed their frustration with their living environment and their helplessness toward it. Community mobilization as a means of health promotion and education has been commonly used by many government and non-government organizations, therefore mobilization without logistically improving the circumstances of the communities no longer amuses rural communities nor does it effectively translate into changing community behaviors.

*“Just like the CoMIC team, people come to us all the time. We sit together and they give us instructions, but we all are poor people, and no one gives us anything. Not everyone can build washrooms on their own.” (FGD, Community Elder, Mobilization Cluster)*

*“Only meetings are going on, but we need resources. We need proper infrastructure in our rural areas. We need streets and gutters.” (FGD, Community Elder, Mobilization Cluster)*

*“The day before yesterday, an entire community refused vaccination. They complained that they do not have anything to eat, and everyday people only come to them for vaccination and polio drops.” (IDI, Teacher, Mobilization Cluster)*

Evidence from the incentive cluster demonstrated that tangible benefits can motivate change in the community’s behaviors. It provided individuals with an opportunity to experiment and experience the advantages of different practices associated with vaccination, hygiene and sanitation, infant and young child feeding (IYCF), and disease management.

*“The benefit to us from your project is that previously people were less interested in vaccines, but now that you have given something that they needed, they have become interested in vaccines.” (FGD, HCPs, Incentive Cluster)*

## Discussion

The community in rural TMK has poor socioeconomic backgrounds with limited infrastructure and access to healthcare. The formative study identifies that the healthcare access in TMK is constrained by many factors, including a shortage of healthcare staff, narrow-focused health education, overburdening of LHWs, and distrust in government facilities [15]. The CoMIC trial recognizes that these systemic gaps affect timely and equitable care for common childhood illnesses. Therefore, the intervention uses an integrated model of Community Mobilization and Conditional, Collective, Community-based Incentives (C3I), which places the community at the center. The communities in rural Sindh have a close-knit social structure, religious and cultural diversity, and a strong sense of collective growth. The formative phase of the CoMIC trial provided a detailed overview of the community’s context in rural TMK, based on which the trial was executed [15]. This study, conducted as a part of the summative phase, evaluates the effectiveness of different components of the CoMIC trial and offers insights that can serve the design of the potential expansion of the trial.

The trial engaged community members, local leaders, and healthcare providers in the design and execution of trial interventions [13,21–23]. Our formative results also showed a strong interest in forming groups for collective action to improve societal behaviors [15]. Evidence shows that community involvement thrives on the interplay between four motives: egoism, altruism, collectivism, and principlism [24]. Each motive is identified by a unique ultimate goal. For egoism, the ultimate goal in community engagement is to seek personal validation; for altruism, to increase the welfare of others; for collectivism, to desire the welfare of a group; and for principlism is to act morally [25–29]. These motives can also be observed in community engagement processes in the CoMIC trial [15,30].

At the same time, initial hesitation was observed, as some community members feared incentives might be delayed or not delivered. This uncertainty created doubts about engaging in the VC process. However, through repeated meetings and transparent discussions, trust was gradually built, and communities became satisfied with their role in decision-making.The bottom-up approach of the trial allowed the community agency to take part in decisions concerning themselves. Although community members strive for a common good, different members of the community may be driven by their own unique reasons and goals. Some of these goals can be instrumental, while others can have unintended consequences [25]. Community engagement as an intervention shows an elaborate combination of various motives and goals coming into play [16]. Rural communities, like TMK, are socially cohesive. Therefore, there is a strong desire for social recognition. People are motivated to volunteer and willingly take up leadership roles in community engagement to contribute to the welfare of the community [31,32]. The VCs comprise people of diverse ages, genders, professions, and statuses, which brings many personal and collective motivations into play [33]. For instance, the union councilor, the smallest administrative unit in the local government body, depends on his people to vote for him into a position. Similarly, various village elders contested for social recognition to sustain their decision-making power in their community. Women as VC members developed a sense of empowerment and improvement in social status [34,35]. For these groups, distinct personal goals play a role in showing participation in trial activities [36]. In addition, the opportunity to network, build connections with the project team, and a sense of entitlement in being part of a social change were driving forces for many individuals, especially the VCs who went an extra mile to ensure compliance through one-on-one counseling and household visits.

Facilitating change in health behaviors and practices is at the heart of community engagement. However, to achieve behavioral change in public health, community engagement acts as a delivery mechanism, where the community members deliver the targeted health message [13]. Most of the time, community engagement as a delivery mechanism requires a form of governance to manage local affairs and facilitate health information dissemination [37]. The VC served as a governing body whose members were part of the community, which is why the community readily trusted the health-promotion activities led by the VC. Overall, an improvement in the uptake of health practices around disease management of diarrhea and pneumonia was observed. Moreover, improvement in community knowledge on IYCF, immunization, hygiene, and childhood disease management enhanced the community’s confidence and empowered them to take initiative on their own [13]. This is in stark contrast to the results of the formative study, which revealed varied perceptions regarding breastfeeding, complementary feeding, immunization, and ORS usage. The economic challenges faced by the community, such as daily wage dependence, limited their focus solely to immediate survival. This concern for immediate survival overshadowed preventive measures, with healthcare often sought only in emergencies [15]. Altogether, egoism in the form of social recognition and desire to build a positive reputation was commonly found among the communities, especially among those in power [38]. Elements of altruism and collectivism were also reflected in the community’s wish for collective progress [28,39]. Likewise, principlism can be seen in the VCs’ efforts to fairly distribute the incentives [40].

Worldwide, many interventions in the WASH sector have had limited sustainability [41–43]. This is attributed to the acute levels of poverty, lack of knowledge about hygiene, deeply ingrained unhygienic behavior and practices, and weak local government systems. During the formative phase of the trial, the community reported poor water quality, including discolored and foul-smelling water. Sanitation conditions were also poor, with a significant proportion of the population practicing open defecation due to the lack of proper toilet facilities. Handwashing practices were inadequate, often performed without soap, which exacerbated the spread of diseases [15]. Thus, the CoMIC trial did not merely focus on service delivery of WASH services; instead, it equipped the community with skills to build and manage the WASH services [44–46]. The decision-making process for selecting WASH facilities prioritized community needs and fairness. Incentives served as an initial push and were phased out as families continued healthy practices due to observed health benefits, supported by links to government services, ensuring sustainability. Families were initially motivated by incentives, but behavioral change communication and community discussions reinforced the value of these practices, helping sustain motivation beyond the incentives. Installation of hand pumps and toilets was done with consensus from the community. Health information sessions further facilitated behavioral change to improve access to WASH services [13,46,47]. To ensure lasting benefits of the provided WASH facilities, the project team and the community members shared the costs by 75 and 25 percent respectively [16]. The monetary contribution and efforts made by the community in the form of labor and land encouraged the community members to take the incentive or service more seriously [48,49]. Among the range of options available, the community put serious thought into shortlisting one WASH facility that they collectively deemed important. This places a psychological factor among the people enhancing the community’s likelihood to avail and take care of the provided facility as it is the community itself that has chosen the facility, and its location, and has shared the cost of its construction [13,50]. Equity concerns also emerged, as some households feared exclusion from incentives. In response, transparent targeting criteria and open meetings ensured that decisions were collectively agreed upon, which enhanced perceptions of fairness.Community mobilization and incentivization have influenced the health behaviors of people in TMK [13,51]. The qualitative findings indicate improvements in knowledge, attitude, and practices of people on the matters of exclusive breastfeeding, complementary feeding, immunization, hygiene, and prevention and management of childhood diarrhea and pneumonia [51]. Consistency was observed among all the interviewed caregivers (Table 6) in terms of health information with some caregivers being more proactive in application than others. The caregivers often recalled and problematized their previous maternal and child health practices in comparison to the newly learned health information. However, acceptance of and adherence to different health interventions or practices varied. For instance, behavioral change towards IYCF practices gained more acceptability than immunization, as health system level challenges, such as access to and availability of vaccines, and individual level challenges, such as low awareness and vaccine hesitancy, still prevail. The changes in IYCF practices were crucial as there was minimal evidence of exclusive breastfeeding (EBF) during the baseline study, with evidence showing that only 4 out of every 10 babies in Pakistan were exclusively breastfed [15,52]. Similarly, ingrained lifestyle behaviors and practices have made it difficult for people to fully utilize some WASH services. For instance, although open defecation has decreased due to the building of latrines and washrooms, the use of these services has only become more gendered. In some cases, women find it shameful to use washrooms in the presence of male members in the house which restricts women’s use of washrooms [53–55]. In other cases, men continue to practice open defecation as they work outside the homes which also restricts men’s use of washrooms [56]. There is a need for awareness and enhanced infrastructural change to facilitate behavioral change in this regard [10,57,58].

This summative research assessed the impact of community engagement and community-based incentivization on the uptake and adherence to recommended preventive and curative practices for diarrhea and pneumonia. Furthermore, the findings contributed to the existing literature on behavioral change interventions intending to inform and inspire future behavioral change and communications interventions [59,60].. Lastly, the summative research has provided an opportunity to identify the conditions and adaptations necessary for successful implementation of the CoMIC approach in different settings. These include strong community structures to support collective decision-making, transparent mechanisms to ensure equitable distribution of incentives, and alignment with existing health system resources and capacities. Adaptations may also be required to respond to variations in cultural norms, socioeconomic conditions, and levels of health service availability across districts or provinces. At the policy level, the findings emphasize the importance of integrating community-led incentive models into district health planning and budgeting frameworks, with the potential to inform national strategies on community engagement and behavior change interventions for child health.

## Limitation

While the study offers valuable insights into how community-driven mobilization and incentivization strategies influence child health behaviors in resource-constrained settings, certain limitations should be noted. The findings are based on self-reported perceptions and behaviors, which may be influenced by recall and social desirability biases, and some reporting bias cannot be excluded despite triangulation across respondent groups. The purposive sampling design may have limited inclusion of marginalized voices, such as religious minorities, seasonal laborers, or those with minimal interaction with Village Committees, affecting the breadth of perspectives. Data collection conducted shortly after the CoMIC trial (December 2020–March 2021) constrained the ability to assess long-term sustainability and motivation once incentives were withdrawn. Additionally, a broader systems-level analysis of policy, financial, and governance factors was beyond the scope of this qualitative design.

## Conclusion

The CoMIC trial in rural Pakistan demonstrated that collective community engagement and incentivization can positively influence child health behaviors. Improvements were most evident in IYCF practices, while progress in immunization and WASH was moderated by vaccine hesitancy, gendered constraints, and infrastructural barriers. The identification of community engagement motives egoism, altruism, collectivism, and Principlism together with evidence of community cost-sharing and ownership, highlights the potential for sustaining behavior change beyond the project’s duration. At the policy level, the findings support integrating community-led incentive models into district health planning with consideration for scale-up in other resource-limited rural settings.

## Supporting information

S1 File Checklist. (DOCX)
